# Use of Machine Learning Techniques for Fertilization Traceability Discrimination via Core Quality Indicators of Korla Fragrant Pear Fruits

**DOI:** 10.3390/foods15112003

**Published:** 2026-06-04

**Authors:** Junkai Zeng, Haixia Wang, Mingyang Yu, Yan Chen, Jianping Bao

**Affiliations:** 1 College of Horticulture and Forestry Science, Tarim University, Alar 843300, China; zjk19520008580@126.com (J.Z.); wanghaixia2026@126.com (H.W.); yumingyangxj@126.com (M.Y.); 10757232053@stumail.taru.edu.cn (Y.C.); 2 Southern Xinjiang Special Fruit Trees High-Quality, High-Quality Cultivation and Deep Processing of Fruit, Products Processing Technical National Local Joint Engineering Laboratory, Alar 843300, China

**Keywords:** korla fragrant pear, machine learning, fruit quality indicators, fertilization traceability

## Abstract

Rational fertilization directly affects the fruit quality of the Korla fragrant pear. However, the variation patterns of fruit appearance and texture indicators under different N-P_2_O_5_-K_2_O ratios are complex, and redundancy among high-dimensional indicators restricts the practical application of quality discrimination and fertilization traceability. In this study, Korla fragrant pear fruits harvested under eight fertilization treatments (including the control) were selected as research materials. Significant differences existed in nutrient composition and application rate among treatments: no N-P_2_O_5_-K_2_O was applied in the CK treatment; for treatments H1–H7, nitrogen (N) application rate ranged from 396.36 to 524.2 g·plant^−1^, phosphorus (P_2_O_5_) from 326.08 to 652.17 g·plant^−1^, and potassium (K_2_O) from 450.67 to 1200.08 g·plant^−1^, with the most prominent differences observed in P-K ratios and application rates. On this basis, 12 appearance and flesh texture indicators were determined, including single-fruit weight, longitudinal diameter, transverse diameter, fruit shape index, pericarp thickness, sclereid content, hardness, adhesiveness, cohesiveness, springiness, gumminess and chewiness. Three machine-learning algorithms, namely Random Forest (RF), Extreme Learning Machine (ELM) and K-Nearest Neighbor (KNN), were used to construct fruit quality discriminant models. The results showed that the RF model achieved the optimal discriminative performance, with accuracy values of 0.876 and 0.865 for the training and validation sets, respectively. Seven core quality indicators, including sclereid content and longitudinal diameter, were screened via feature-importance intersection analysis. The reconstructed RF model based on this indicator set exhibited nearly no loss in discriminative accuracy despite a ~42% reduction in indicator quantity, providing theoretical and technical support for quality grading, fertilization traceability and precision fertilization of Korla fragrant pear.

## 1. Introduction

Korla Fragrant Pear (*Pyrus sinkiangensis Yu*), an elite indigenous pear cultivar in southern Xinjiang, is widely popular for its thin peel, crisp flesh, abundant juice and sweet taste [1]. As a characteristic fruit for both fresh consumption and processing, its quality stability and traceability directly affect consumer acceptance and industrial competitiveness [2]. Southern Xinjiang features a typical temperate, extreme continental arid climate with low soil organic matter and intense evaporation. Improper fertilization not only wastes resources but also deteriorates fruit quality [3]. N-P-K ratio is a key cultivation factor regulating vegetative growth and fruit quality of pear trees, yet systematic studies are lacking on its comprehensive effects on the appearance and texture properties of Korla Fragrant Pear. Different from previous studies focusing on other pear cultivars or conventional indicators such as yield and soluble solids [4,5,6].

Existing studies mostly adopt traditional statistical methods to evaluate the effects of fertilization rate and ratio on yield and conventional quality indicators (e.g., soluble sugar content, titratable acidity, single-fruit weight) of Korla Fragrant Pear. However, such methods fail to reveal the complex nonlinear relationships between quality indicators and fertilization treatments, nor can they effectively screen core quality indicators most sensitive to fertilization responses [7]. In contrast, machine-learning approaches require no predefined functional forms and better adapt to nonlinear and strongly interactive data characteristics commonly observed in agricultural systems [8]. In recent years, machine learning has exhibited great potential in quality discrimination and traceability research of agricultural products [9]. Random Forest (RF) can handle high-dimensional data and assess feature importance [10]. Extreme Learning Machine (ELM) features fast training speed and strong generalization ability [11]. K-Nearest Neighbor (KNN) is intuitive, with no explicit training required [12]. Multi-model feature screening can overcome the selection bias of a single model and extract stable core indicators most sensitive to fruit quality variations under different fertilization regimes [13]. Nevertheless, previous studies generally relied on a single model (e.g., RF or partial least-squares discriminant analysis), which easily causes unstable indicator selection due to model structural differences [14]. In this study, an intersection analysis of three heterogeneous models (RF, ELM, KNN) was applied to significantly improve the robustness of core indicator screening.

Accordingly, this study collected Korla Fragrant Pear fruits under different N-P-K ratio treatments and measured 12 appearance and texture indicators, including single-fruit weight, longitudinal diameter, transverse diameter, fruit shape index, pericarp thickness, stone cell content, hardness, adhesiveness, cohesiveness, springiness, gumminess and chewiness. Three discrimination models (RF, ELM, KNN) were constructed to evaluate their capacity in distinguishing fruit quality differences corresponding to various fertilization backgrounds. Furthermore, core quality features consistent across models were screened via feature-importance intersection analysis, and the model performance with simplified features for fruit discrimination was validated. This study aims to provide data-driven methodological support for rapid quality grading, fertilization source tracing and post-harvest quality evaluation of Korla Fragrant Pear, as well as a reference for developing quality traceability techniques for characteristic fruits in arid regions. By systematically assessing 12 appearance and texture traits specifically for Korla Fragrant Pear, this research fills the gap in the multi-dimensional quality evaluation of this cultivar in response to N-P-K ratios.

## 2. Materials and Methods

### 2.1. Experimental Materials and Site

**Figure 1:**
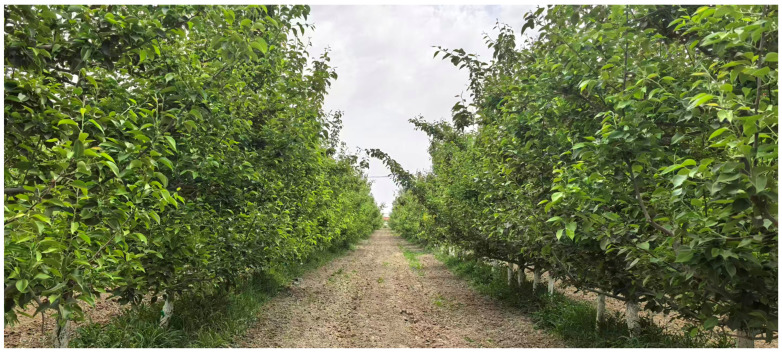
Experimental site.

Field experiments were conducted on 20 September 2024 and 20 September 2025 at Lihua Town, Aral City, Xinjiang Uygur Autonomous Region, China (40°33′14″ N, 81°0′40″ E, Figure 1). The experimental site lies on the northern bank of the upper Tarim River at an average altitude of 1017 m, featuring a typical temperate extreme continental arid climate with annual precipitation of approximately 50 mm. The soil is sandy-loam with strong surface evaporation, abundant solar-thermal resources and large diurnal temperature differences.

The plant materials were 5-year-old densely planted trunk-trained Korla Fragrant Pear trees grafted on Pyrus betulaefolia rootstocks. The orchard was established in late March 2016, with grafting completed in late March 2017. Trees were planted in north–south rows with a spacing of 1.5 m × 4 m under a central-leader training system. Basic soil physicochemical properties are shown in Table 1.

### 2.2. Experimental Design

Based on conventional fertilization practices for Korla Fragrant Pear in the Aksu region of Xinjiang, a single-factor completely randomized block design was adopted. The mass ratios of phosphorus (P_2_O_5_) and potassium (K_2_O) were set as independent variables, while nitrogen (N) was fixed at a baseline level (N = 1) in most treatments. The phosphorus gradient in treatments H1–H3 was achieved by synchronously adjusting N and P application rates. Twelve appearance and texture quality indicators of Korla Fragrant Pear were taken as dependent variables to investigate the effects of different N-P-K nutrient ratios on fruit quality.

Eight fertilization treatments were established (Table 2), including a non-fertilized control (CK) and seven pure-nutrient mass-ratio treatments (H1–H7) of N-P_2_O_5_-K_2_O. P_2_O_5_ and K_2_O ratios served as the core independent variables, whereas N was maintained at a constant baseline level (non-gradient variable) across most treatments. The 12 dependent variables were single-fruit weight, longitudinal diameter, transverse diameter, fruit shape index, pericarp thickness, stone cell content, hardness, adhesiveness, cohesiveness, springiness, gumminess and chewiness.

The N:P_2_O_5_:K_2_O ratios of each treatment were 1:0.5:1 (H1), 1:0.75:1 (H2), 1:1:1 (H3), 1:0.5:0.75 (H4), 1:0.5:1.5 (H5), 1:0.5:1.75 (H6), and 1:0.5:2 (H7). These ratios refer to the mass ratio of pure nutrients N-P_2_O_5_-K_2_O. The values presented in the table represent the application rates of pure nutrients, rather than the actual mass of commercial fertilizers. Urea (46% N) and diammonium phosphate (18% N) were used as nitrogen sources; diammonium phosphate (46% P_2_O_5_) served as the phosphorus source; and potassium sulfate (51% K_2_O) was selected as the potassium source. The specific application rates of N, P_2_O_5_, and K_2_O for each treatment are shown in Table 2.

Fertilization was performed at the pre-bud-burst stage, young-fruit expansion stage and rapid-fruit-expansion stage, with 30%, 40% and 30% of the total nutrient rate applied at each stage, respectively. Nitrogen fertilizer was urea (N ≥ 46%), phosphorus fertilizer was diammonium phosphate (P_2_O_5_ ≥ 46%), and potassium fertilizer was potassium sulfate (K_2_O ≥ 50%). Fertilizers were applied in annular furrows 20–30 cm deep, followed by soil covering and irrigation.

Each treatment included three biological replicate plots, with three uniformly growing pear trees selected per plot, resulting in 72 experimental trees in total. Plots were arranged in a randomized block design with guard rows between them. Flood irrigation was conducted once in early June, July and August, respectively.

### 2.3. Sample Collection and Pretreatment

Fruits from each fertilization treatment were harvested at the ripening stage of Korla Fragrant Pear (20 September 2024 and 20 September 2025). Ten pest- and damage-free fruits were collected from the east, south, west, and north directions of each tree, labeled, transported to the laboratory, and stored in a refrigerator at 4 °C for subsequent analysis.

### 2.4. Determination of Fruit Appearance Quality

Fifty fruits were randomly selected from each fertilization treatment and the control group, with a total of 400 fruits used for appearance-quality measurement. Five indicators were individually determined for each fruit with three replicates, and the mean value per fruit was used for subsequent statistical analysis.

**Figure 2:**
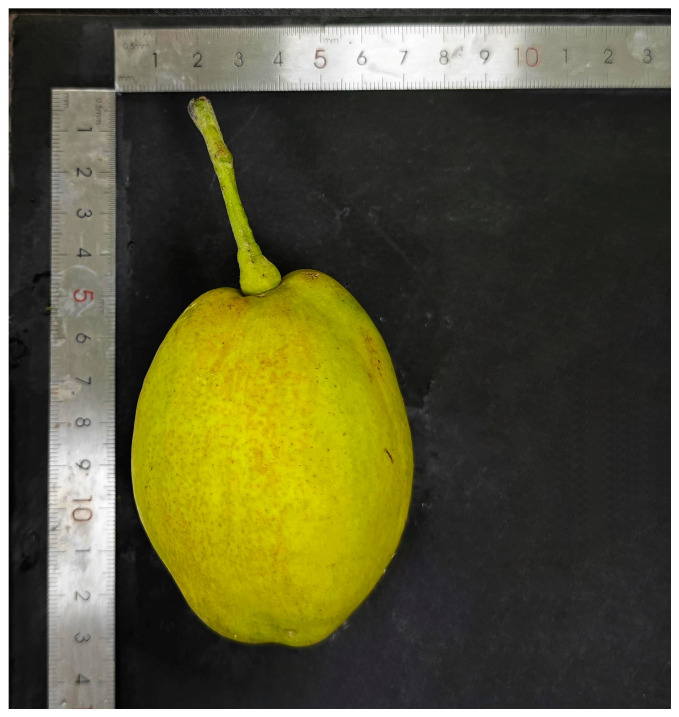
Morphological characteristics of the tested Korla Fragrant Pear fruits.

Single-fruit weight was measured using an electronic balance (precision: 0.01 g). Longitudinal and transverse diameters were determined with a digital vernier caliper (precision: 0.01 mm). The longitudinal diameter referred to the maximum vertical distance from the stem-end base to the calyx-end apex, and the transverse diameter represented the maximum equatorial diameter. The fruit shape index was calculated as the ratio of longitudinal diameter to transverse diameter. For pericarp thickness, two 5 mm × 5 mm peel segments (without flesh) were sampled symmetrically on both sides of the fruit equator; the middle thickness of each segment was measured, and the average of the two measurements was recorded per fruit. All fruits were measured following the above procedure, and data from each treatment were summarized separately [15]. The measured sample is shown in Figure 2.

### 2.5. Determination of Fruit Texture

Fifty fruits per treatment (including the control), totaling 400 fruits, were used to determine stone cell content and fruit texture properties (hardness, adhesiveness, cohesiveness, springiness, gumminess, chewiness), with three replicates per fruit.

Stone cell content was measured using the freezing-hydrochloric acid separation method. Pulp samples were taken from different equatorial positions of each fruit, mixed, and 10.0 g (accurate to 0.01 g) of the mixed sample was weighed, cut into pieces, and boiled in 100 mL of 1.0 mol/L HCl for 30 min. After cooling, the suspension was filtered through a 200-mesh sieve and rinsed with distilled water until neutral. Stone cells retained on the sieve were dried to constant weight at 80 °C, and their percentage relative to fresh pulp weight was calculated [16].

Fruit texture properties were determined by texture profile analysis (TPA) using a texture analyzer (TA.XT Plus, Stable Micro Systems, Godalming, UK) [17]. After peeling each fruit, three 10 mm × 10 mm × 8 mm pulp cylinders were cut from the equatorial region. Test parameters were set as follows: P/50 probe, pre-test speed 2.0 mm/s, test speed 1.0 mm/s, post-test speed 1.0 mm/s, compression depth 40% of pulp thickness, trigger force 5 g, and a 5 s interval between two compressions. All 12 fruit quality indicators (Table 3) were measured strictly in accordance with the national standard GB/T 10650-2008 [18] Fresh Pears and the general TPA testing specifications for pomaceous fruits. Hardness (maximum peak force of the first compression, N), adhesiveness (negative peak area of the first compression, N·s), cohesiveness (ratio of positive peak areas of the second to the first compression), and springiness (height recovery ratio before the second compression) were derived from force-time curves. Gumminess (N) = hardness × cohesiveness; chewiness (N·mm) = hardness × cohesiveness × springiness. The average of three measurements per pulp cylinder was taken as the representative value for each fruit. All 400 fruits were measured following the above procedures, and data for each treatment were statistically analyzed separately.

### 2.6. Data Standardization Processing

To eliminate the effects of dimensional differences among indicators on model performance, raw data were standardized using the Z-score method [19], which normalizes each indicator to a mean of 0 and a standard deviation of 1. The standardization formula is shown in Equation (1).

(1) $$x_{ij}^{scaled}=\frac{x_{ij}-\bar{x_{j}}}{\sigma_{j}}$$

where $x_{ij}$ = raw value of the i-th sample for the j-th indicator; $\bar{x_{j}}$ = mean value of the j-th indicator; $\sigma_{j}$ = standard deviation of the j-th indicator.

### 2.7. Dataset Partitioning

All 400 samples were stratified and randomly split into a training set and a validation set at a ratio of 7:3. Stratified sampling was applied to ensure that the proportion of each fertilization treatment in both datasets was consistent with the original data [8]. A random seed of 42 was set during splitting to guarantee result reproducibility.

### 2.8. Machine Learning Model Construction

#### 2.8.1. Extreme Learning Machine

ELM is a single-hidden-layer feedforward neural network. Its input weights and hidden layer biases are randomly initialized, and output weights are analytically calculated using the least squares method. In this study, the number of hidden-layer nodes in the ELM model was set to 1000, and the activation function was the Sigmoid function. ELM exhibits fast training speed and strong generalization ability, making it suitable for processing high-dimensional small-sample data [20].

#### 2.8.2. k-Nearest Neighbors

KNN is an instance-based non-parametric classification algorithm. It calculates the distance between the sample to be classified and each sample in the training set, selects the K nearest neighbors, and assigns the class by majority voting. In this study, Euclidean distance was used as the distance metric, the optimal K-value (K = 5) was determined by grid search, and inverse-distance weighting was applied for feature weights [21].

#### 2.8.3. Random Forest

RF is an ensemble learning algorithm based on decision trees. Multiple training subsets are generated via Bootstrap sampling, and decision trees are constructed for each subset; the final classification result is obtained through majority voting. In this study, the parameters of the RF model were set as follows: number of decision trees (n_estimators) = 200, maximum depth (max_depth) = 10, minimum number of samples per leaf (min_samples_leaf) = 2, maximum number of features (max_features) = sqrt(n_features), and the Gini coefficient was used as the splitting criterion [22].

### 2.9. Model Evaluation Metrics

To predict the fertilization treatment category to which each sample belongs, four commonly used metrics in machine learning classification evaluation were adopted to assess model performance in this study: Accuracy (Equation (2)), Precision (Equation (3)), Recall (Equation (4)), and F1 Score (Equation (5)) [9]. Meanwhile, to evaluate the fitting precision between the predicted and measured values, two additional metrics were introduced: mean squared error (MSE, Equation (6)) and multiple correlation coefficient (R, Equation (7)), which were used to assess the model’s accuracy and explanatory power. The calculation formulas for each metric are presented as follows:

(2) $$\mathrm{Accuracy}\;=\frac{\mathrm{TP}+\mathrm{TN}}{\mathrm{TP}+\mathrm{TN}+\mathrm{FP}+\mathrm{FN}}$$

(3) $$Precision=\frac{TP}{TP+FP}$$

(4) $$Recall=\frac{TP}{TP+FN}$$

(5) $$F1=2\times \frac{Precision\times Recall}{Precision+Recall}$$

where TP denotes true positives, referring to the number of samples correctly classified into the corresponding category by the model; TN denotes true negatives, the number of non-target samples correctly identified as non-target by the model; FP denotes false positives, non-target samples incorrectly classified into the target category; FN denotes false negatives, target samples incorrectly classified as non-target samples.

(6) $$MSE=\frac{1}{n}\sum_{i=1}^{n}{\left(y_{i}-\hat{y_{i}}\right)}^{2}$$

(7) $$R=\sqrt{\frac{\sum_{i=1}^{n}{\left(\hat{y_{i}}-\bar{y}\right)}^{2}}{\sum_{i=1}^{n}{\left(y_{i}-\bar{y}\right)}^{2}}}$$

In Equation (6), $y_{i}$ is the measured value of the i-th sample, $\hat{y_{i}}$ is the predicted value of the model, and n is the total number of samples. A smaller MSE value indicates a smaller deviation between the predicted and measured values of the model.

In Equation (7), $\bar{y}$ is the mean of all measured data. An R value closer to 1.0 indicates a better fitting effect of the model on the measured data.

In this study, the criteria for determining the optimal state of the model were defined as follows: the mean squared error (MSE) should be reduced to the order of 10^−4^, and the multiple correlation coefficient (R) should infinitely approach 1.0. A smaller MSE indicates a smaller deviation between predicted and measured values; a multiple correlation coefficient closer to 1 indicates a better overall fitting performance of the model (Table S2).

### 2.10. Construction of Feature Evaluation, Screening and Discrimination Models

#### 2.10.1. Assessment of Feature Importance

Built-in feature importance evaluation methods of each model were adopted in this study. For RF, the Mean Decrease in Gini was used [23]; for ELM, the absolute values of connection weights from the input layer to the output layer were applied [24]; and for KNN, recursive feature elimination (RFE) combined with cross-validation was utilized [25].

#### 2.10.2. Core Feature Screening

To eliminate algorithmic bias introduced by single-model feature evaluation and obtain key quality indicators with higher stability and universality, a Venn diagram was used to analyze the intersection of high-importance features screened by the three machine-learning models (RF, ELM, and KNN) [26]. The detailed procedures were as follows. First, indicators with an importance score ≥ 7 were extracted separately from RF, ELM, and KNN models to form three individual feature sets. Second, pairwise and triple intersection operations were performed on the three sets using a Venn diagram tool. Finally, indicators common to the high-importance feature sets of all three models were defined as the “core feature set”. Features selected by this method show stable discriminative contributions across different algorithm structures and effectively avoid feature misselection caused by overfitting or specific data distribution in a single model, thereby providing more reliable and simplified input variables for subsequent discriminant model construction.

#### 2.10.3. Construction of the Discrimination Model for Korla Fragrant Pears

Based on the core feature set filtered by the intersection of the three models in Section 2.10.2, the discriminative model is constructed by using the model algorithm that performs best in the construction of the full feature algorithm. Taking the indicators in the core feature set as input variables and eight different fertilization treatments (CK, H1–H7) as output labels, the training set and validation set were divided according to the stratified random sampling method described in Section 2.7 (training set: validation set = 7:3, random seed 42). The model hyperparameter Settings are consistent with the full feature model in Section 2.8. After completing the model training on the training set, the model performance is evaluated, and the discrimination results are compared and analyzed with those of the model based on 12 full feature indicators to verify the representativeness of the core feature set.

### 2.11. Data Analysis

The original data of all fruit quality indicators in this study were preliminarily sorted out and summarized using Microsoft Excel 2021. Descriptive statistical analysis (mean, standard deviation, coefficient of variation) and bar chart plotting were completed in Origin 2021 software (OriginLab Corporation, Northampton, MA, USA) The distribution characteristics of indicators such as single fruit weight, longitudinal diameter, transverse diameter, fruit shape index, fruit peel thickness, stone cells, hardness, adhesion, cohesion, elasticity, adhesiveness and chewiness of each treatment group are presented. The construction and evaluation of machine learning models (including data standardization, hierarchical division of training sets/validation sets, training of RF/ELM/KNN models, hyperparameter tuning, calculation of feature importance, and calculation of model performance indicators) are all conducted in MATLAB R2024b (MathWorks Inc., Natick, MA, USA). It is implemented in the USA environment by self-writing scripts and invoking the corresponding toolboxes (Statistics and Machine Learning Toolbox, Deep Learning Toolbox) [27].

## 3. Results and Analysis

### 3.1. Distribution Characteristics of Pear Fruit Appearance Quality Under Different Fertilization Treatments

**Figure 3:**
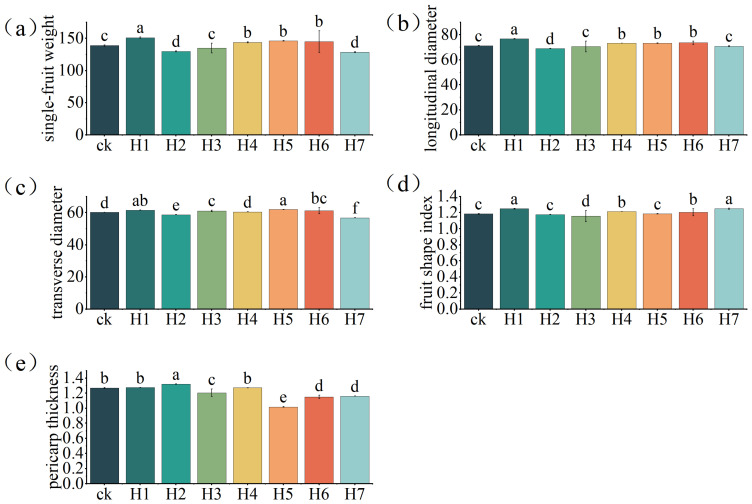
Changes in appearance quality of pear fruits under different fertilization treatments: (**a**) Single-fruit weight; (**b**) Longitudinal diameter; (**c**) Transverse diameter; (**d**) Fruit shape index; (**e**) Pericarp thickness. Different lowercase letters indicate significant differences at *p* < 0.05 by Duncan’s multiple range test.

To clarify the effects of different N-P-K ratios on the appearance quality of Korla fragrant pear fruits, seven fertilization treatments (H1–H7) were set with CK (no fertilization) as the control. Five indicators, including single-fruit weight, longitudinal diameter, transverse diameter, fruit shape index and pericarp thickness, were measured respectively. The mean values (±standard deviation) of each treatment are shown in Figure 3. Overall, fertilization ratios significantly altered fruit appearance quality. Among them, fruit shape index and pericarp thickness were the most sensitive to treatment variations, whereas transverse diameter exhibited extremely high consistency across all treatments.

Different N-P_2_O_5_-K_2_O fertilization ratios exhibited significantly differentiated regulatory effects on core appearance quality indicators of Korla fragrant pear, including single-fruit weight, longitudinal and transverse diameters, fruit shape index and pericarp thickness (*p* < 0.05). Appearance indicators showed regular responses to gradient changes in phosphorus and potassium ratios, which could be used for traceability discrimination of fruit quality under different fertilization regimes.

Single-fruit weight was the highest under treatment H1 (N:P_2_O_5_:K_2_O = 1:0.5:1), which was significantly higher than that under other treatments (Figure 3a,b). Under fixed potassium application rates (H1–H3), single-fruit weight decreased with increasing phosphorus proportion. When the phosphorus rate was fixed and potassium proportion increased gradually (H1, H4–H7), single-fruit weight first increased and then decreased with elevated potassium supply, remaining at high levels in H4–H6 and declining significantly in H7. These results indicated that low-potassium and moderate-phosphorus ratios could significantly improve fruit size, whereas excessive potassium inhibited single-fruit weight accumulation.

Fruit longitudinal diameter reached its maximum under H1 (N:P_2_O_5_:K_2_O = 1:0.5:1), which was significantly greater than that of other treatments. Under fixed potassium levels (H1–H3), longitudinal diameter continuously declined with increasing phosphorus proportion. With a fixed phosphorus supply and gradually increased potassium proportion (H1, H4–H7), longitudinal diameter maintained high values in H4–H6 but decreased significantly under H7. Overall, low-potassium and moderate-phosphorus ratios significantly promoted longitudinal fruit growth, while excessive phosphorus or potassium inhibited longitudinal diameter development. Significant differences in longitudinal diameter among fertilization treatments could distinguish various phosphorus-potassium supply patterns, providing quantitative evidence of longitudinal growth for fertilization traceability discrimination of Korla fragrant pear.

Fruit transverse diameter generally increased with rising potassium proportion, with the maximum value observed under H6 (1:0.5:1.75) (Figure 3c). Among phosphorus-gradient treatments H1–H3, H1 showed the largest transverse diameter, and excessive phosphorus was unfavorable to transverse diameter development. The fruit shape index reflected morphological differences among fruits; H1 and H7 possessed significantly higher fruit shape indices, while H4 and H5 produced relatively round fruits (Figure 3d). Fertilization ratios directly altered the fruit morphology of Korla fragrant pear by regulating longitudinal and transverse diameter development, which could serve as morphological evidence for fertilization traceability.

Pericarp thickness was markedly regulated by fertilization ratios, being the thickest under H2 and the thinnest under H5 (Figure 3e). A clear pattern was observed: high phosphorus increased pericarp thickness, whereas high potassium reduced it. H5 and H6 had the thinnest pericarp (approximately 1.017 mm), H1 exhibited an intermediate thickness (1.275 mm), and high-phosphorus treatments showed the thickest pericarp. In summary, phosphorus and potassium ratios directionally regulated core appearance indicators of Korla fragrant pear in this study. Significant differences in appearance indicators across fertilization treatments provided quantitative morphological evidence for traceability discrimination of fertilization regimes.

### 3.2. Distribution Characteristics of Pear Fruit Parenchyma Under Different Fertilization Treatments

**Figure 4:**
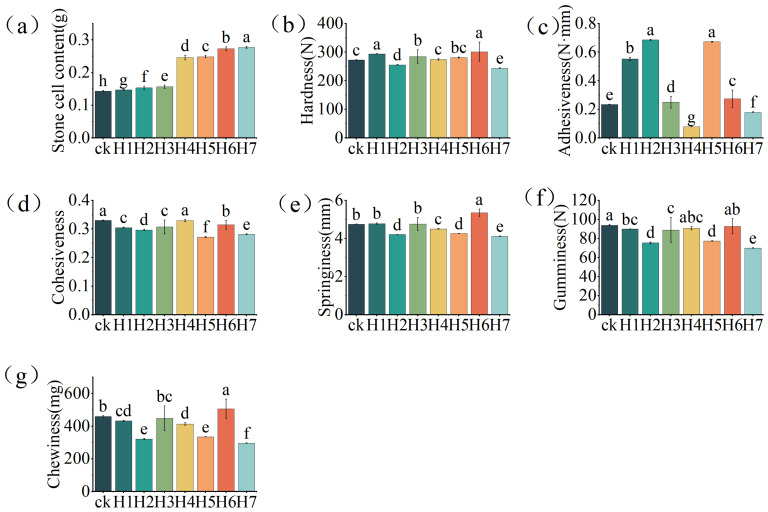
Changes in texture quality of pear fruits under different fertilization treatments: (**a**) Stone cell content; (**b**) Hardness; (**c**) Adhesiveness; (**d**) Cohesiveness; (**e**) Springiness; (**f**) Gumminess; (**g**) Chewiness. Note: Different lowercase letters indicate significant differences in the same indicator among different fertilization ratios (*p* < 0.05).

To investigate the effects of different N-P_2_O_5_-K_2_O ratios on pear fruit texture, six indicators, including stone cell content, hardness, adhesiveness, cohesiveness, springiness and gumminess, were measured in this study (Figure 4). Bar charts of 50 replicates (mean ± standard deviation) under each treatment (CK and H1–H7) showed that fertilization ratios significantly altered most texture parameters. Among them, stone cell content, hardness and adhesiveness were the most sensitive to treatment responses, while cohesiveness exhibited negligible differences across treatments with high inherent varietal stability.

Comprehensive comparison of the six texture indicators under all treatments (CK and H1–H7) revealed that different N-P_2_O_5_-K_2_O fertilization ratios exerted significant regulatory effects on stone cell content and flesh texture characteristics of Korla fragrant pear (*p* < 0.05). Overall, increasing potassium (K_2_O) proportion significantly increased stone cell content, hardness, springiness and chewiness, whereas excessive potassium reduced flesh cohesiveness and gumminess. Phosphorus supply moderately improved the stability of flesh texture.

Stone cell content continuously increased with rising potassium proportion in fertilization ratios (Figure 4a), with the highest value under H7 (N:P_2_O_5_:K_2_O = 1:0.5:2), which was significantly higher than those of other treatments. Stone cell content slightly increased with increasing phosphorus proportion in H1–H3, while the non-fertilized control CK had the lowest stone cell content. These results indicated that potassium fertilizer was the key factor inducing stone cell accumulation, phosphorus had a relatively mild effect, and different potassium levels could directly distinguish fertilization patterns.

Overall, flesh hardness first increased and then decreased with elevated potassium proportion (Figure 4b), peaking under H5 (1:0.5:1.5) and declining significantly under H7. Among phosphorus-gradient treatments H1–H3, H5 showed the maximum hardness, which decreased with increasing phosphorus proportion. This suggested that moderate potassium application improved flesh firmness, excessive potassium reduced flesh compactness, and excessive phosphorus was unfavorable for hardness maintenance.

Adhesiveness was most significantly affected by fertilization ratios (Figure 4c), being significantly highest under H2 and H5, and lowest under H4. The basic adhesiveness of CK was relatively low, indicating that appropriate phosphorus-potassium ratios improved flesh adhesiveness, whereas insufficient potassium markedly reduced flesh viscosity. Adhesiveness presented a bimodal distribution: significantly higher in H1–H3 and H5, while lower than CK in H4 and H7. This indicator was extremely sensitive to N-P-K ratios, yet the effects of excessively high or low values on sensory quality need further verification combined with sensory evaluation. Cohesiveness showed minimal differences among all treatments (maximum difference = 0.059), presenting high conservatism. It was not suitable as an indicator for evaluating fertilization effects but could serve as a varietal background reference for pear fruit texture and a sensitive discriminant indicator for distinguishing phosphorus-potassium supply differences.

Cohesiveness was generally highest under CK and H4 (Figure 4d) and decreased significantly with a continuous increase in potassium proportion (H5–H7), reaching the minimum under H7. Springiness and chewiness showed consistent variation patterns (Figure 4e,g), both peaking under H6 (1:0.5:1.75). Springiness and chewiness under phosphorus-gradient treatments H1–H3 were generally lower than those under low-potassium treatments. Gumminess first increased and then decreased with rising potassium proportion (Figure 4f), with optimal values under H4 and H5 and a significant decline under H7.

In summary, phosphorus played a weak regulatory role in texture indicators, whereas potassium acted as the dominant regulator. Moderate potassium application improved hardness, springiness and chewiness, but excessive potassium caused massive stone cell accumulation, decreased cohesiveness and deteriorated edible quality of flesh. Significant and regular differences in stone cell and texture indicators among fertilization treatments, combined with appearance indicators, could be used to construct a fertilization traceability discrimination system for Korla fragrant pear.

### 3.3. Construction and Evaluation of Discrimination Models for Different Fertilization Treatments Based on Machine Learning Algorithms

**Figure 5:**
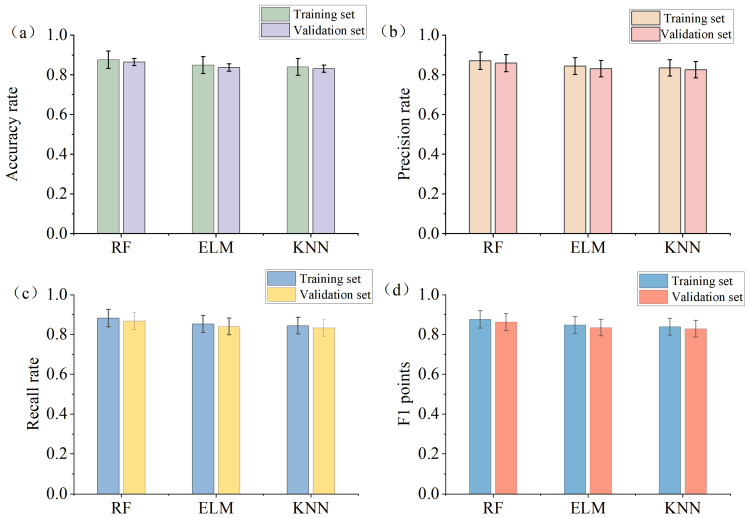
Construction and evaluation of discriminant models for eight different fertilization treatments based on three algorithms (RF, ELM, KNN). (**a**) Accuracy; (**b**) Precision; (**c**) Recall; (**d**) F1-Score.

To explore the feasibility of rapidly distinguishing different fertilization treatments (CK, H1–H7) based on fruit quality indicators, we took fruit appearance quality and texture profile data as input features and constructed discriminant models using three algorithms: Random Forest (RF), Extreme Learning Machine (ELM) and K-Nearest Neighbor (KNN). Model performance was evaluated on the training set and validation set. The results are presented in Figure 5 and Tables S4–S7 in the Supplementary Materials.

Overall, all three models could effectively distinguish different fertilization treatments, with RF exhibiting the optimal performance across all evaluation indicators. On the training set, RF achieved an accuracy of 0.876, higher than that of ELM (0.849) and KNN (0.840) (Table S1). On the validation set, the accuracy values of RF, ELM and KNN were 0.865, 0.837 and 0.831, respectively. The accuracy differences between the training and validation sets were all less than 2%, indicating no obvious overfitting for any of the three models, among which RF performed superiorly.

Further comparisons of precision, recall and F1-score also revealed the superiority of RF. For the training set, the precision, recall and F1-score of RF were 0.871, 0.882 and 0.876, respectively, while those on the validation set were 0.859, 0.868 and 0.863. The corresponding indicators of ELM and KNN were slightly lower; for instance, the F1-score of ELM and KNN on the validation set was 0.835 and 0.829, respectively. For a fair comparison of the three models, confusion matrices of RF, ELM and KNN were generated based on the validation set (Figure S1), and Matthews Correlation Coefficient squared (MCC^2^) values were calculated accordingly (Table S2). The results demonstrated that RF maintained the highest values for all indicators with the smallest decline between the training and validation sets, suggesting that the Random Forest algorithm can better capture the complex nonlinear relationships between fruit quality characteristics and fertilization treatments.

### 3.4. Core Quality Index Identification Based on Multi-Model Feature Screening

**Figure 6:**
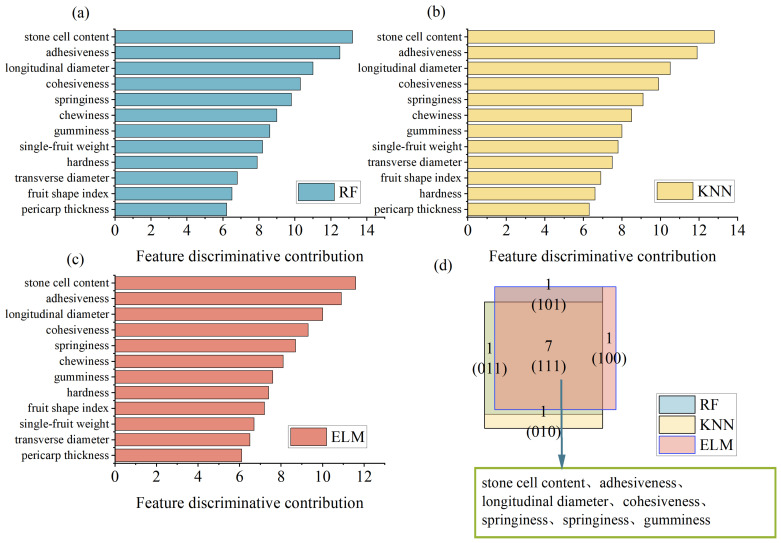
Feature extraction results and feature intersection analysis based on three models. (**a**) Features and their contribution degrees of the RF model; (**b**) Features and their contribution degrees of the KNN model; (**c**) Features and their contribution degrees of the ELM model; (**d**) Feature intersection analysis.

To identify fruit quality indicators closely associated with fertilization treatments and screen those with high discriminative ability for fertilization categories, three machine-learning algorithms, including Extreme Learning Machine (ELM), K-Nearest Neighbor (KNN) and Random Forest (RF), were used to select core quality indicators with stable discriminative capacity. The results are shown in Figure 6. In feature screening, the importance threshold was set to 7, and only indicators with importance scores no less than this value were retained as core features to ensure high contribution and reliability for fertilization treatment discrimination.

According to the feature importance distributions of each model (Figure 6a–c), differences existed among high-importance indicators. In the RF model (Figure 6a), stone cell content, adhesiveness, longitudinal diameter, cohesiveness, springiness, chewiness, gumminess, single-fruit weight and hardness showed significantly higher importance scores than other indicators. In the KNN model (Figure 6b), stone cell content, adhesiveness, longitudinal diameter, cohesiveness, springiness, chewiness, gumminess, single-fruit weight and transverse diameter were identified as high-contribution features. For the ELM model (Figure 6c), stone cell content, adhesiveness, longitudinal diameter, cohesiveness, springiness, chewiness, gumminess, hardness and fruit shape index were listed as the most important features.

To eliminate selection bias from a single model and further improve the stability and universality of screening results, intersection analysis of high-importance indicators from the three models was conducted (Figure 6d). Seven core features were commonly screened by all three models, namely stone cell content, adhesiveness, longitudinal diameter, cohesiveness, springiness, chewiness and gumminess. These indicators had importance scores ≥ 7 and ranked high in all models, demonstrating stable discriminative performance across different algorithm structures and acting as key quality indicators for distinguishing different fertilization treatments in this study.

In summary, stone cell content, adhesiveness, longitudinal diameter, cohesiveness, springiness, chewiness and gumminess can serve as core input features for the fertilization-treatment discriminant model of Korla fragrant pear. This feature set provides a reliable database for precise evaluation of fertilization schemes and regulation of fruit quality.

### 3.5. Construction and Mechanism Analysis of a Discrimination Model for Fertilization Treatment of Korla Fragrant Pears Based on RF

**Figure 7:**
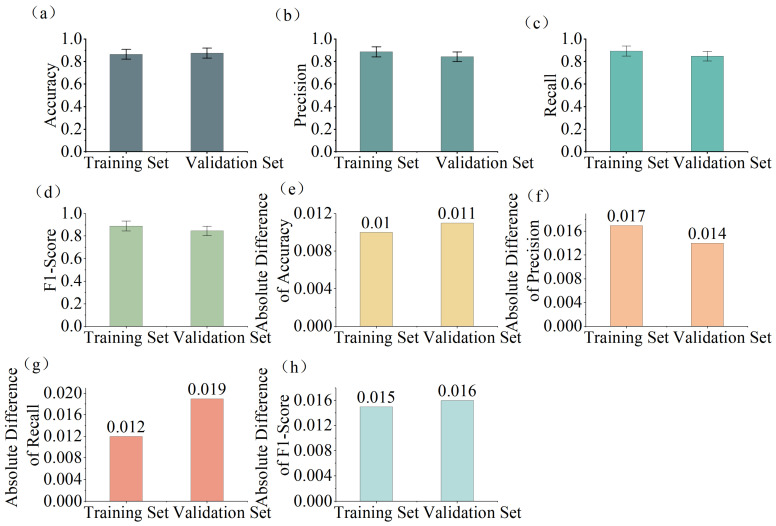
Model validation of the seven-feature model and comparative analysis with the full-feature model based on the RF algorithm. (**a**) Accuracy of the RF-7-feature model; (**b**) Precision of the RF-7-feature model; (**c**) Recall of the RF-7-feature model; (**d**) F1-Score of the RF-7-feature model; (**e**) Absolute difference in accuracy; (**f**) Absolute difference in precision; (**g**) Absolute difference in recall; (**h**) Visualization of absolute difference in F1-Score.

Based on seven core features screened from the intersection of three machine-learning models (stone cell content, adhesiveness, longitudinal diameter, cohesiveness, springiness, chewiness and gumminess), a discriminant model for fertilization treatments of Korla fragrant pear was constructed and systematically evaluated, with results shown in Figure 7. Performance evaluation of the seven-feature model (Figure 7a–d) revealed excellent and stable discriminative ability on both training and validation sets: the training-set accuracy, precision, recall and F1-score were approximately 0.866, 0.888, 0.894 and 0.891, respectively, while the corresponding validation-set metrics were about 0.876, 0.845, 0.849 and 0.847. No significant differences were observed between the two datasets, indicating no overfitting and good generalization ability of the model.

Difference analysis between the full-feature model and seven-feature model (Figure 7e–h) further compared their evaluation metrics. The absolute differences in accuracy, precision, recall and F1-score for the seven-feature model were 0.01, 0.017, 0.012 and 0.015 on the training set and 0.011, 0.014, 0.019 and 0.016 on the validation set, all at extremely low levels. These results demonstrated that the core-feature model greatly reduced input dimensionality (from 12 to 7 features) with nearly no performance loss, achieving an optimal balance between model complexity and discriminative accuracy.

## 4. Discussion

Korla fragrant pear fruits harvested under different N-P_2_O_5_-K_2_O ratios exhibited considerable differences in appearance and flesh texture properties. In this study, 12 fruit quality indicators were measured, and machine-learning models were established to explore which indicators best reflect quality differentiation under different fertilization backgrounds, and a stable and low-redundancy core quality indicator system was screened out.

### 4.1. The Physicochemical Basis of Fruit Quality Differentiation Caused by Fertilization Background

Changes in fruit appearance and texture are ultimately determined by how N-P-K supply regulates cell division, cell expansion, cell wall metabolism and accumulation of secondary metabolites. Different N-P-K fertilization ratios induce significant differentiation in appearance and internal texture quality of Korla fragrant pear by regulating fruit cell morphological development, cell wall structure, stone cell formation and substance metabolism of flesh texture. Their physicochemical basis is mainly reflected in four aspects: cell structure construction, cell wall component metabolism, stone cell development and texture substance accumulation, which also serve as the internal mechanism for screening core traceability indicators in this study.

N-P-K ratios regulate cell expansion and morphological development, driving differentiation in appearance quality. In this experiment, treatments H1–H3 represented fixed potassium (1) with increasing phosphorus gradients (N:P_2_O_5_:K_2_O = 1:0.5:1, 1:0.75:1, 1:1:1, respectively), whereas H1 and H4–H7 represented fixed phosphorus (0.5) with increasing potassium gradients (K = 0.75–2). Treatment H1 (balanced N-P-K ratio of 1:0.5:1) exhibited the significantly highest single-fruit weight and longitudinal diameter. With increasing phosphorus proportion (H1→H2→H3), single-fruit weight as well as longitudinal and transverse diameters declined, indicating that excessive phosphorus inhibited fruit cell expansion. Under fixed phosphorus supply, transverse diameter gradually increased with elevated potassium levels (H4→H1→H5→H6→H7), while excessively high potassium (H7) disrupted cell turgor balance, inhibited late-stage fruit expansion and reduced single-fruit weight. Potassium also regulated pericarp cell wall thickness, ultimately resulting in morphological differences in fruit size, shape and pericarp thickness among fertilization regimes, which formed the morphological basis for using appearance indicators in fertilization traceability.

In terms of fruit texture, stone cell content directly determines the fineness of pear fruit taste [28]. Potassium dominated stone cell differentiation and lignin deposition, acting as the core physicochemical mechanism underlying texture quality differentiation. Under fixed phosphorus levels, stone cell content continuously increased with rising potassium proportion (H4–H7), reaching the maximum under H7. This result was consistent with the findings of Feng et al. on Huangguan pear that potassium application increased pectin content and fruit firmness [29], while Hasan M.U. et al. reported that potassium deficiency led to thinner pericarp, and high leaf phosphorus was correlated with thin pericarp and low acidity in ‘Kinnow’ mandarin [30]. By contrast, phosphorus gradients (H1–H3) exerted weak effects on stone cells, demonstrating that potassium was the core nutrient inducing stone cell accumulation in pear flesh. Massive stone cell formation increased flesh hardness, springiness and chewiness, whereas excessive stone cells destroyed the continuity of flesh structure and reduced cohesiveness and gumminess. Moderately high-potassium ratios in H5 and H6 (1:0.5:1.5–1.75) balanced stone cell development and flesh texture, achieving optimal comprehensive texture performance. Therefore, stone cell content was the most sensitive physicochemical indicator of fertilization-induced quality differentiation and a core traceability feature screened by machine learning in this study.

Adhesiveness fluctuated most drastically across all treatments: it was markedly higher in H2, H3 and H5 but extremely low in H4. Adhesiveness is mainly governed by the bonding force of pectin in flesh [31]. Previous studies have shown that potassium application increases pectin content, and the calcium-potassium ratio is critical for quality indicators such as firmness and soluble solids content [32]. Phosphorus participates in the synthesis and metabolism of pectin and cellulose; the balanced ratio of H1 maintains cell wall integrity. Potassium regulated cell wall bonding properties by modulating the activities of pectinase and cellulase, thereby controlling adhesiveness and gumminess. Treatments H2 (1:0.75:1, high-phosphorus balanced potassium) and H5 (1:0.5:1.5) had appropriate P-K ratios with vigorous pectin metabolism, resulting in significantly the highest flesh adhesiveness. Low potassium (H4) or excessively high potassium (H7) disrupted pectin metabolism and decreased adhesiveness. Stable differences in cell wall substance metabolism among fertilization regimes endowed texture indicators such as hardness, springiness and chewiness with traceability potential.

Cohesiveness showed almost no differences among all treatments. It measures the ability of flesh to resist internal cracking and is highly associated with varietal genetic background [33]. Early studies have noted that accumulation patterns of storage substances at physiological maturity exert certain effects on cohesiveness, yet fertilization barely alters it [34]. Such high consistency across treatments, conversely, makes cohesiveness a valuable reference indicator. Although it cannot be used to evaluate fertilization effects, it can verify sample stability among batches. Overall, different N-P-K ratios systematically modified fruit morphology and texture by affecting potassium-calcium balance, PAL activity and pectin metabolism. Treatment H1 achieved a favorable balance in multiple dimensions, whereas high phosphorus or extremely high potassium caused obvious negative effects on certain quality attributes.

N-P-K regulated the steady state of fruit physiological metabolism through nutrient balance, leading to differentiated responses of quality indicators. Excessive phosphorus (H3) and excessively high potassium (H7) caused nutrient imbalance, disturbed normal cell metabolism and aggravated abnormal stone cell development. The balanced ratio of H1 (1:0.5:1) maintained metabolic equilibrium and best facilitated fruit expansion. Regular responses of appearance and texture indicators to fertilization ratios in this study were essentially external manifestations of nutrient-regulated cell structure and substance metabolism. Appearance morphology, stone cell content and key texture indicators jointly characterized differences in fertilization backgrounds, providing a solid physicochemical basis for fertilization traceability discrimination of Korla fragrant pear.

### 4.2. Comparison of the Ability of Machine Learning Models to Distinguish Differences in Fruit Quality

All three models could effectively distinguish fruit samples from different fertilization backgrounds using fruit quality indicators, yet the RF model outperformed ELM and KNN in terms of accuracy, precision, recall and F1-score. This result is consistent with the inherent mechanisms of the three algorithms. As a tree-based algorithm, RF constructs multiple decision trees via Bootstrap sampling and random feature subspace strategies, enabling it to automatically capture nonlinear interactions among fruit quality indicators (e.g., the negative correlation between stone cell content and hardness, or the effect of coordinated changes in longitudinal and transverse diameters on fruit shape index). Consequently, RF achieved the best performance on the high-dimensional and small-sample dataset in this study. These findings are basically in agreement with previous studies [35,36].

The performance differences between training and validation sets for all three models were less than 2%, indicating no overfitting occurred. This suggests that fruit quality indicators themselves contain sufficient information to differentiate various fertilization backgrounds, without requiring complicated feature engineering. Although RF showed the most outstanding performance in this study, model adaptability should be flexibly evaluated according to data characteristics in practical applications.

### 4.3. The Food Science Significance and Cross-Model Stability of Core Quality Indicators

The seven core indicators screened by feature-importance intersection analysis (stone cell content, adhesiveness, longitudinal diameter, cohesiveness, springiness, chewiness and gumminess) all have clear physicochemical correlations with the edible quality and processing suitability of pear fruits. Stone cells are formed by lignin deposition in sclerenchyma cells, and their content directly determines the mouthfeel roughness of flesh, acting as a unique texture-degrading factor in pears [37]. Five textural parameters, including adhesiveness, cohesiveness, springiness, chewiness and gumminess, reflect the mechanical behaviors of flesh during compression deformation, fracture resistance, deformation recovery and energy dissipation from different dimensions, which collectively form the physical basis for fresh-eating taste and processing properties of pear fruits (e.g., juice yield and jam viscosity) [38]. As a simple geometric indicator, longitudinal diameter significantly affects fruit shape classification and commercial grading, and is associated with postharvest packaging and transportation damage risks [39]. The screening results of the above indicators reveal that fertilization treatments mainly affect fruit edible quality and processing suitability rather than simply fruit size [40].

High-importance feature sets differed among the three models. RF assigned high weights to single-fruit weight and hardness, which represent total dry-matter accumulation and cell wall fracture resistance, respectively. KNN was sensitive to transverse diameter because, as a key parameter reflecting fruit flatness, it possessed high discriminative power under Euclidean distance measurement. ELM paid more attention to fruit shape index (longitudinal diameter/transverse diameter), a ratio that eliminates the influence of absolute fruit size and purely expresses geometric morphology. Such inter-model differences reflect algorithm-specific preferences for feature response patterns, whereas the seven indicators obtained from their intersection exhibit cross-algorithm stability.

Cohesiveness showed minimal numerical differences among fertilization treatments (maximum difference = 0.059, standard deviation ≤ 0.01), yet all three models assigned it high importance. This result is not anomalous but derived from its physicochemical properties: cohesiveness characterizes the ability of flesh to resist internal micro-fractures, which is mainly controlled by cell-wall polysaccharide cross-linking structures and varietal genetic background, and is insensitive to external nutrient disturbances [41]. Precisely due to its extremely low variation across treatment groups (high signal-to-noise ratio), cohesiveness serves as a stable anchor for discriminating different fertilization backgrounds in classification models [42]. Although cohesiveness does not reflect fertilization effects, its high cross-treatment consistency and low measurement noise endow it with the properties of a high-quality discriminative feature [43]. From the perspective of fruit quality evaluation, this characteristic enables cohesiveness to act as a baseline reference for correcting batch-to-batch sample variations, facilitating the establishment of more stable quality grading standards [44].

### 4.4. The Significance of Simplifying the Core Indicator Set for Rapid Evaluation of Fruit Quality

The RF discriminant model reconstructed using the seven core indicators exhibited nearly identical performance to the full-indicator model (absolute differences: 0.010–0.019). Similar findings have been verified in quality discrimination studies of other fruits. For instance, Kim et al. reported that wavelength selection greatly reduced input dimensionality while maintaining high prediction accuracy when forecasting soluble solid content in citrus fruits [45]. Wang et al. achieved optimal prediction of passion fruit exocarp hardness using only seven characteristic wavelengths, combining near-infrared spectroscopy with feature wavelength selection methods such as CARS and SPA [46]. These studies further confirm that simplified feature combinations produce satisfactory discriminative performance in practical applications.

These indicators are highly correlated with fruit edible quality and can be used as target traits for high-quality fruit cultivation, as well as for post-harvest grading and fertilization-background traceability. Notably, cohesiveness showed minimal variation among treatments in this study, yet its high consistency across treatments makes it a valuable reference for evaluating sample stability. This provides a noteworthy new perspective for the selection of fruit quality evaluation indicators.

### 4.5. Research Limitations and Prospects

This study still has some deficiencies. Firstly, the fruit sampling was only from one planting site and two consecutive years, without including different climate year types or soil types. Therefore, the extrapolation of the established core index set and discriminant model needs further verification. Secondly, the method for determining the content of stone cells belongs to destructive chemical separation and is not suitable for non-destructive testing. In the future, it is possible to explore the use of near-infrared spectroscopy technology to establish a rapid prediction model for the content of stone cells, thereby achieving non-destructive or minimally destructive detection of all seven indicators. In addition, among the seven core indicators of this study, there are multiple fruit texture parameters. However, the specific corresponding relationship between them and consumers’ sensory evaluations is still unclear. Subsequently, the weight of the indicators can be further optimized by combining sensory assessment data to make the discrimination results closer to consumers’ actual feelings.

## 5. Conclusions

This study systematically evaluated the differences in 12 appearance and texture indicators of Korla fragrant pear fruits harvested under different N-P_2_O_5_-K fertilization ratios. The results of difference analysis among treatment groups, together with the mean and standard deviation of each fruit quality indicator, are summarized in Supplementary Table S3. Three machine learning algorithms, namely RF, ELM and KNN, were adopted to establish discriminant models based on fruit quality indicators. The main conclusions are presented as follows:

The Random Forest model achieved the optimal performance in fruit quality discrimination, with training-set and validation-set accuracies of 0.876 and 0.865, respectively, outperforming ELM and KNN. No obvious overfitting was observed, indicating that fruit quality indicators can effectively reflect the effects of different fertilization backgrounds.

Seven cross-model consistent core fruit quality indicators were screened out via multi-model feature-importance intersection analysis: stone cell content, adhesiveness, longitudinal diameter, cohesiveness, springiness, chewiness and gumminess. These indicators are directly related to the eating taste, processing suitability and commercial grading of pear fruits, possessing clear food-science significance.

Compared with the full-indicator model, the RF discriminant model built on the seven core indicators had absolute differences below 0.02 in accuracy, precision, recall and F1-score. This demonstrates that drastically reducing detection indicators causes no practical loss in discriminative accuracy, providing a feasible indicator set for rapid fruit quality evaluation and fertilization-background traceability.

Comprehensively considering fruit appearance and texture characteristics, fruits harvested from treatment H1 (N:P_2_O_5_:K_2_O = 1:0.5:1) exhibited the largest single-fruit weight, highest hardness, moderate stone cell content and attractive fruit shape. Thus, H1 is a preferable fertilization ratio for improving the quality of Korla fragrant pear.

The core fruit quality indicator set and discriminant model established in this study can provide methodological support for post-harvest grading, quality traceability and standardized evaluation of high-quality Korla fragrant pear. They also offer a referable technical route for quality discrimination research of other characteristic fruits.

## Figures and Tables

**Table 1:** Basic physicochemical properties of orchard soil.

Measured Index	Soil Depth (cm)		
	0~20	20~40	40~60
pH	8.08	8.07	8.13
Alkali-hydrolyzable N (mg·kg^−1^)	23.65	19.38	15.91
Available P (mg·kg^−1^)	12.19	16.70	4.59
Available K (mg·kg^−1^)	15.65	19.85	12.60

**Table 2:** The application amount of NPK for each fertilization treatment ^1^.

Experimental Unit	N:P_2_O_5_:K_2_O	N (g·Plant^−1^)	P_2_O_5_ (g·Plant^−1^)	K_2_O (g·Plant^−1^)
CK	-	0	0	0
H1	1:0.5:1	524.2	326.09	600.55
H2	1:0.75:1	460.44	489.13	600.55
H3	1:01:01	396.36	652.17	600.55
H4	1:0.5:0.75	524.2	326.08	450.67
H5	1:0.5:1.5	524.2	326.08	900.32
H6	1:0.5:1.75	524.2	326.08	1050.2
H7	1:0.5:2	524.2	326.08	1200.08

^1^ Ratios refer to mass ratios of pure nutrients (N-P_2_O_5_-K_2_O). Values in the table represent pure-nutrient application rates, not the physical mass of commercial fertilizers.

**Table 3:** Details of the 12 fruit quality indicators measured in this study.

Index Types	Name of Specific Indicator	Desirable Optimal State
Appearance index	Single-fruit weight	The larger the better (higher commercial value)
	Longitudinal diameter	Moderate is optimal (too large/small reduces appearance quality)
	Transverse diameter	Moderate is optimal (to match longitudinal diameter for uniform shape)
	Fruit shape index	The closer to 1, the better (a rounder shape improves marketability)
	Pericarp thickness	The thinner the better (improves edible quality)
	Stone cell content	The lower the better (lower content means finer flesh texture)
Texture indicators	Hardness	Moderately high (balances storability and eating quality)
	Adhesiveness	The lower the better (reduces sticking during consumption)
	Cohesiveness	Moderate (ensures stable flesh structure without brittleness)
	Springiness	Moderately high (higher springiness indicates crisp texture)
	Gumminess	The lower the better (reduces chewing difficulty and stickiness)
	Chewiness	Moderate (balances crispness and ease of mastication)

## Data Availability

The original contributions presented in the study are included in the article/Supplementary Materials; further inquiries can be directed to the corresponding author.

## Supplementary Materials

The following supporting information can be downloaded at: https://www.mdpi.com/article/10.3390/foods15112003/s1, Figure S1: Confusion matrices of discriminant models for eight fertilization treatments based on RF, ELM and KNN algorithms; Table S1: Average classification performance of different machine learning models on the validation set. Table S2: Comparison of category discrimination performance indicators among the three models; Table S3: Mean and standard deviation of fruit quality indices under different treatments; Table S4: Analysis of variance for model performance; Table S5: ANOVA test results of each indicator; Table S6: Correlation coefficient matrix of performance indicators; Table S7: Comparison of model generalization ability.

## Abbreviations

The following abbreviations are used in this manuscript:

RF	Random Forest
ELM	Extreme Learning Machine
KNN	K-Nearest Neighbor
CK	Control Check
TPA	Texture Profile Analysis
RFE	Recursive Feature Elimination
NPK	Nitrogen, Phosphorus, Potassium
