# Supplementary material for: Use of Machine Learning Techniques for Fertilization Traceability Discrimination via Core Quality Indicators of Korla Fragrant Pear Fruits
Source: Foods. 2026 Jun 4;15(11):2003. doi: 10.3390/foods15112003 (PMC13256611; doi:10.3390/foods15112003)
Supplement: Supplementary file 1 [file foods-15-02003-s001.zip › foods-4329766-supplementary.pdf]

Supplementary File

1.In this study, no separate test set was divided. The validation set (30% of total data) was used to evaluate the generalization performance of the models. Table 1 presents the average classification performance (Accuracy, Precision, Recall and F1-score) of three machine learning models including RF, ELM and KNN on the validation set.

Table S1. Average classification performance of different machine learning models on the validation set

Model	Accuracy	Precision	Recall	F1 Score
RF	0.865	0.859	0.868	0.863
ELM	0.837	0.831	0.84	0.835
KNN	0.831	0.826	0.833	0.829

2.We calculated the Matthews Correlation Coefficient (MCC) for each model via the confusion matrix. The squared value of MCC (MCC2) was used as the quadratic correlation coefficient to screen the model with the optimal fitting effect for Figure 7.

It should be noted that Figure 7 only presents the results of the Random Forest (RF) model. To enable a fair comparison across the three models, we constructed the confusion matrices of RF, ELM and KNN based on the validation set (Figure 1), and calculated their corresponding coefficient of determination (R2) and root mean square error (RMSE). The results are presented below.

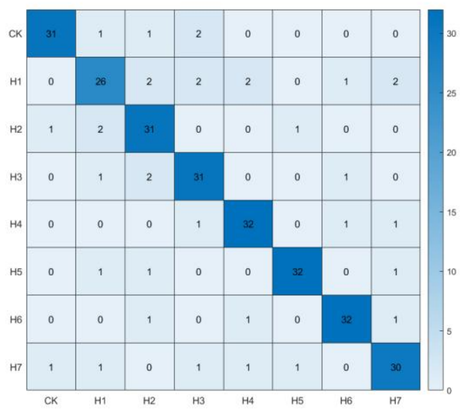

(A)

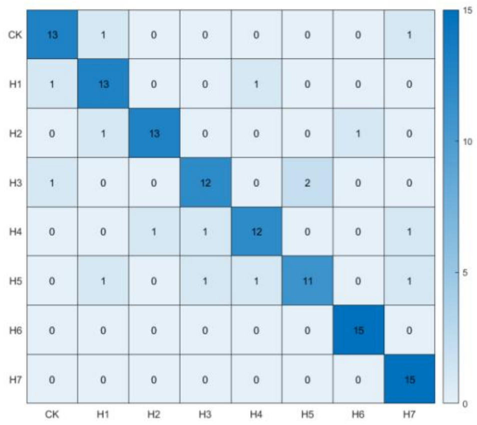

(B)

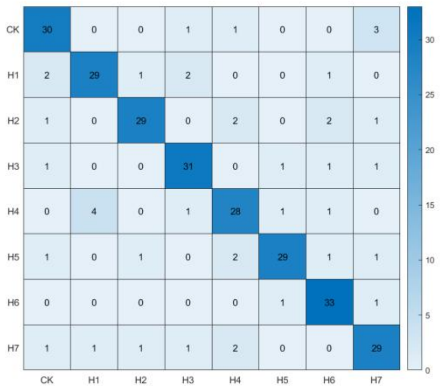

(C)

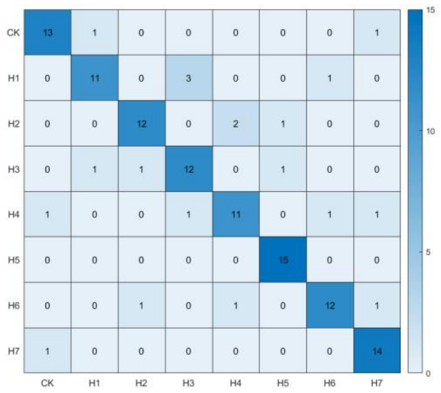

(D)

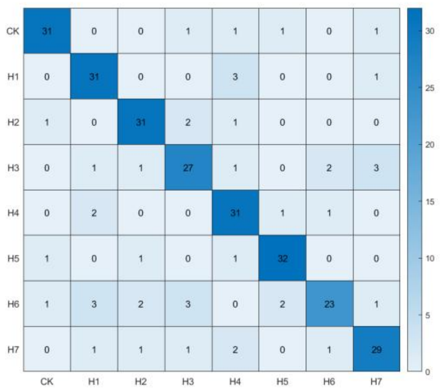

(E)

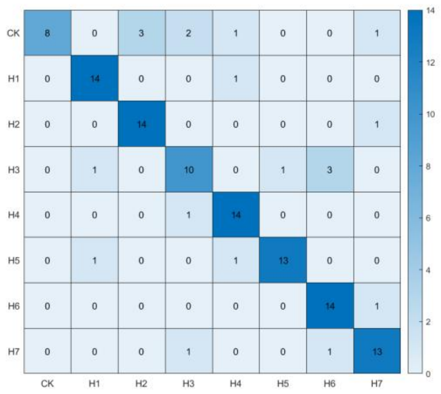

(F)

Figure S1 Confusion matrices of discriminant models for eight fertilization treatments based on RF, ELM and KNN algorithms. (A) Training set confusion matrix of RF; (B) Validation set confusion matrix of RF; (C) Training set confusion matrix of ELM; (D) Validation set confusion matrix of ELM; (E) Training set confusion matrix of KNN; (F) Validation set confusion matrix of KNN.

3.On the basis of clarifying the classification accuracy of each model, we further quantified the discrimination performance and prediction error characteristics of different models for each fertilization treatment group. The discrete labels of fertilization categories were converted into continuous numerical values. The mean square error (MSE), root mean square error (RMSE) and coefficient of determination (R^2) between predicted and actual categories were systematically calculated for Random Forest (RF), Extreme Learning Machine (ELM) and K-Nearest Neighbor (KNN). The evaluation results of each model on the training and validation sets are presented in the table below.

Table S2 Comparison of category discrimination performance indicators among the three models

**Model**	**Dataset**	RMSE	MSE	R^2	**Classification Accuracy**
RF	Training set	1.124	1.263	0.773	0.876
RF	Validation set	1.053	1.109	0.789	0.865
ELM	Training set	1.394	1.943	0.630	0.849
ELM	Validation set	1.332	1.774	0.662	0.837
KNN	Training set	1.428	2.039	0.612	0.821
KNN	Validation set	1.301	1.693	0.678	0.792

Overall comparison shows that all three classification models achieve low prediction errors and good fitting consistency, among which the RF model presents the best comprehensive performance. For the RF model, the RMSE values of the training set and validation set are 1.124 and 1.053, and the MSE values are 1.263 and 1.109, respectively. The low error values indicate that misclassifications mainly occur between adjacent fertilization treatment groups, with no extreme cross-category deviations. Its coefficients of determination (R^2) reach 0.773 and 0.789 for the training and validation sets, demonstrating a strong linear consistency between predicted and true categories as well as excellent discriminative stability.

In contrast, the ELM and KNN models yield higher errors and lower fitting degrees. Specifically, the RMSE and MSE of the KNN training set are 1.428 and 2.039, and the R^2 of its

validation set is only 0.678, meaning its classification accuracy and stability are inferior to those of the RF model.

Combining the results of classification accuracy, error indicators and R^2, the RF model outperforms ELM and KNN in recognition accuracy, stability and error control for different fertilization treatments. It should be noted that these indicators were calculated based on encoded classification labels to quantitatively evaluate the discriminative performance of the models.

Table S3 Mean and Standard Deviation of Fruit Quality Indices Under Different Treatments

Experimental Unit	Statistic	Stone cell content(%)	Single-fruit weight(g)	Longitudinal diameter (mm)	Transverse diameter(mm)	Fruit shape index	Pericarp thickness(mm)	Hardness(N)	Adhesiveness(N·s)	Cohesiveness	Springiness	Gumminess(N)	Chewiness(mJ)
CK	max	0.148009143	140.5801823	71.81153101	60.19384308	1.193673779	1.283387897	275.8027116	0.238648499	0.332600829	4.769808819	95.872343	474.7169283
	min	0.138887844	136.6437076	70.75415117	60.1456637	1.175833905	1.25559179	270.8567043	0.230389887	0.326896855	4.746709204	92.95137718	449.142788
	Average	0.14342709	138.8102749	71.27233748	60.17108698	1.184494821	1.270123098	272.9100316	0.233546388	0.330073346	4.75640506	94.05567662	460.1402697
	SD	0.001802139	0.856949664	0.23737751	0.010446675	0.003956775	0.005622831	1.13115311	0.001825239	0.001520218	0.005315094	0.607780769	4.903391856
	CV	0.012564844	0.006173532	0.00333057	0.000173616	0.003340475	0.004426997	0.004144784	0.007815318	0.004605698	0.00111746	0.006461925	0.010656298
H1	max	0.150444576	153.7023584	77.28398481	61.74013042	1.256218778	1.277931603	296.1253168	0.582516932	0.307505866	4.872654469	90.33753508	439.2432142
	min	0.14266119	148.8881284	76.22177187	61.25654813	1.23533392	1.272936808	292.6081862	0.533752353	0.300524943	4.71140882	89.72440638	424.1401072
	Average	0.146704537	151.0571257	76.76888774	61.51939549	1.247884512	1.275328893	294.3828642	0.552714821	0.304682999	4.783717302	89.97684955	432.9051898
	SD	0.001844696	0.988888666	0.212499265	0.097745145	0.004079018	0.001277254	0.872730892	0.011961143	0.001656339	0.035103744	0.14238422	3.262974025
	CV	0.012574226	0.006546455	0.002768039	0.001588851	0.003268746	0.00100151	0.002964612	0.021640713	0.00543627	0.007338173	0.001582454	0.007537387
H2	max	0.163807462	131.2917285	69.3239615	58.82859329	1.182319507	1.329837957	258.473766	0.698063854	0.301621693	4.226029144	77.44478345	330.2351772
	min	0.143395567	127.9878382	68.8643033	58.48650279	1.172560413	1.314469912	251.7608809	0.677348072	0.291765343	4.198265135	73.4243577	309.9242072
	Average	0.153379499	129.7802574	69.07012496	58.68773053	1.176910632	1.322217977	255.7691581	0.687143221	0.297003886	4.212751259	75.53258685	321.0536761

Experimental Unit	Statistic	Stone cell content(%)	Single-fruit weight(g)	Longitudinal diameter (mm)	Transverse diameter(mm)	Fruit shape index	Pericarp thickness(mm)	Hardness(N)	Adhesiveness(N·s)	Cohesiveness	Springiness	Gumminess(N)	Chewiness(mJ)
	SD	0.005100186	0.769814932	0.103571744	0.067881674	0.002198909	0.003743823	1.400992697	0.003581106	0.002004682	0.006838583	0.901132976	3.563975812
	CV	0.033252071	0.00593168	0.001499516	0.001156659	0.001868374	0.002831472	0.005477567	0.005211587	0.006749682	0.001623306	0.011930387	0.011100872
	max	0.166509705	152.4710704	77.78245999	62.0619193	1.278014471	1.314263164	342.0035475	0.32977714	0.373330966	5.570134856	111.7852632	613.633521
	min	0.14510901	118.4857222	62.50225353	59.84042704	1.018766567	1.060623005	240.0048972	0.181167101	0.260401767	4.076975497	67.89665405	330.0874157
H3	Average	0.156384672	135.0283283	70.61722142	61.00305511	1.157660485	1.205440141	284.896676	0.24952172	0.30746206	4.764276759	88.9432688	449.0308986
	SD	0.005139174	7.41558991	4.214904195	0.528434558	0.069348169	0.048824187	23.6341672	0.039768277	0.02388773	0.341099175	13.06686153	73.59524456
	CV	0.032862391	0.054918772	0.059686633	0.008662428	0.05990372	0.040503203	0.082956978	0.159378019	0.07769326	0.071595164	0.146912315	0.163897952
	max	0.262347692	145.3018282	73.39139009	60.47473633	1.214064925	1.276943117	280.0565739	0.081233296	0.33996046	4.55820589	94.79861941	435.49309
	min	0.228385474	142.0444513	73.3319309	60.43133635	1.212992424	1.26910404	269.6885764	0.075171691	0.320100437	4.470159752	86.2667303	397.2378165
H4	Average	0.24584019	143.858927	73.36183586	60.45497162	1.213495519	1.272811344	274.8117058	0.078132295	0.329327711	4.506257213	90.83445161	413.6626298
	SD	0.007013948	0.696931644	0.014338477	0.009998306	0.000259714	0.001893391	2.304163966	0.001232274	0.003862775	0.020820206	1.710364618	7.979261403
	CV	0.028530516	0.004844549	0.000195449	0.000165384	0.000214021	0.001487566	0.008384519	0.015771636	0.011729273	0.004620288	0.01882947	0.019289297
	max	0.257417336	146.7173894	73.80187152	62.0871774	1.193068068	1.027173911	284.5880222	0.677016358	0.275311687	4.281913604	77.95993405	337.8895889
H5	min	0.238790852	145.323058	72.93182656	61.8056956	1.176978636	1.005266253	274.2736772	0.667952067	0.26843713	4.253346437	76.95401522	332.257019
	Avera	0.248598353	146.1594447	73.39728183	61.9334688	1.185099779	1.017916558	280.9677	0.672973553	0.272084	4.270426	77.59324	334.6227

Experimental Unit	Statistic	Stone cell content(%)	Single-fruit weight(g)	Longitudinal diameter (mm)	Transverse diameter(mm)	Fruit shape index	Pericarp thickness(mm)	Hardness(N)	Adhesiveness(N•s)	Cohesiveness	Springiness	Gumminess(N)	Chewiness(mJ)
	ge							193		216	635	427	182
	SD	0.003514676	0.302333596	0.191122065	0.057073102	0.003250398	0.005043081	2.015370006	0.002223875	0.001534568	0.006051719	0.21618806	0.96574766
	CV	0.01413797	0.002068519	0.002603939	0.000921523	0.002742721	0.004954316	0.007172959	0.003304551	0.005640047	0.001417123	0.002786171	0.002886079
	max	0.285656797	187.361116	77.11879609	65.79826861	1.297690766	1.199149981	365.6507278	0.389124234	0.353735155	5.849276958	108.4302212	622.4722296
	min	0.262290154	117.4119676	71.03684093	57.39698065	1.120105634	1.103834612	234.1854188	0.172887692	0.28241035	4.892405782	77.39829013	384.4400895
H6	Average	0.273116282	144.8893192	73.68879554	61.25647031	1.20412389	1.150929651	301.5343367	0.27349909	0.314517311	5.355188042	92.89501117	507.0037135
	SD	0.005224729	16.62298182	1.294670093	1.896216251	0.043409996	0.022076133	33.21975218	0.060207051	0.014568242	0.188228875	7.861735119	58.55070761
	CV	0.019130052	0.114728828	0.017569429	0.030955363	0.036051105	0.019181132	0.110169053	0.220136203	0.046319363	0.035148882	0.084630326	0.115483785
	max	0.28340936	130.4355721	71.86088288	56.91759301	1.266972124	1.164168078	246.6966675	0.184736273	0.285204119	4.15768855	70.79406249	297.9505918
	min	0.268826299	126.8471392	70.04315422	56.65106905	1.232731435	1.157220555	241.6094272	0.173834856	0.278067881	4.093277271	69.54226777	292.2203073
H7	Average	0.277281801	128.7017576	70.90244313	56.78439098	1.24862861	1.160962649	244.0136437	0.179764012	0.281906053	4.121376577	70.17482716	295.7326105
	SD	0.003418638	0.829829302	0.411674948	0.063776514	0.007627548	0.001581048	1.243914171	0.002337575	0.00142317	0.013494936	0.305212895	1.209432157
	CV	0.01232911	0.006447692	0.005806217	0.001123135	0.006108741	0.001361843	0.005097724	0.013003576	0.005048386	0.003274376	0.004349322	0.004089614

5.Comprehensive Evaluation of Statistical Tests and Generalization Ability for Model Performance

To clarify the differences and reliability of the classification performance of RF, ELM and KNN models, one-way analysis of variance (ANOVA), indicator correlation analysis and generalization ability comparison were adopted to systematically verify the model performance.

5.1 Analysis of Variance for Model Performance

One-way ANOVA was performed on the overall classification performance of the three models, and the results are shown in Table S4.

Table S4 Analysis of variance for model performance

Source	SS	df	MS	F	Pr> F
Between Groups	0.00797	2	0.003985	84.60	< 0.0001 ***
Within Groups/Error	0.00099	21	0.000047		
Total	0.00896	23			

Extremely significant differences were observed between groups ($F=55.1043$, $p<0.001$), indicating that the classification capability of the three models presented statistically significant genuine differences rather than random errors. The overall data showed small variation and a low sum of squares for within-group errors, which reflected good stability of the experimental results.

Furthermore, one-way ANOVA was conducted separately for four evaluation indicators including accuracy, precision, recall and F1-score (Table S5). The p-values of all indicators were less than 0.05, suggesting that these indicators could effectively distinguish the performance differences among models. Significant differences were found in precision, recall and overall classification ability across models.

Table S5 ANOVA test results of each indicator

**Performance indicator**	**F statistic**	p value	**Significance**
Accuracy	11.7775	0.0380	*
Precision	10.1294	0.0463	*

Performance indicator	F statistic	p value	Significance
Recall	9.6725	0.0492	*
F1 Score	9.5913	0.0497	*

5.2 Correlation Analysis of Evaluation Indicators

To verify the consistency and reliability of the evaluation system, a multiple correlation analysis was conducted on the four classification indicators (Table S6). The results showed that the pairwise correlation coefficients among accuracy, precision, recall and F1 Score were all greater than 0.99, indicating an extremely strong positive correlation. It demonstrates that the multi-dimensional evaluation indicators selected in this study present consistent judgment trends and the evaluation system is highly stable, which further confirms the validity and reliability of the model performance comparison results.

Table S6 Correlation coefficient matrix of performance indicators

Performance indicator	Accuracy	Precision	Recall	F1 Score
Accuracy	1.000	0.9996	0.9987	0.9993
Precision	0.9996	1.000	0.9990	0.9998
Recall	0.9987	0.9990	1.000	0.9996
F1 Score	0.9993	0.9998	0.9996	1.000

5.3 Model Performance Ranking and Generalization Ability Evaluation

Quantitative comparison of model performance was performed based on validation set data. The comprehensive performance ranking is: RF > ELM > KNN. The RF model achieved the optimal overall classification accuracy, with an accuracy of 0.865 and an F1 Score of 0.863. The ELM model ranked second, with accuracy and F1 Score of 0.837 and 0.835, respectively. The KNN model exhibited the weakest overall performance, with an accuracy of 0.831 and an F1 Score of 0.829.

Table S7 Comparison of model generalization ability

Model	Accuracy difference between training set and validation set	Overfitting risk
RF	0.011	Low
ELM	0.012	Moderate
KNN	0.009	Low

As shown in Table S7, the generalization ability and overfitting risk were evaluated via the accuracy difference between the training set and validation set. The RF and KNN models presented tiny accuracy deviations with low overfitting risk and excellent generalization stability. By contrast, the ELM model had a slightly larger deviation and a moderate overfitting risk.

Combining the results of significance test via analysis of variance, indicator correlation verification and generalization ability comparison, the Random Forest model exhibited significantly better overall performance than ELM and KNN. It achieved the highest classification accuracy, most stable prediction performance and optimal generalization ability, thus being the optimal classification model for distinguishing different fertilization treatments in this study.
